# Pain assessment in elderly with dementia: Brazilian validation of the PACSLAC scale

**DOI:** 10.1590/S1679-45082016AO3628

**Published:** 2016

**Authors:** Karol Bezerra Thé, Fernanda Martins Gazoni, Guilherme Liausu Cherpak, Isabel Clasen Lorenzet, Luciana Alves dos Santos, Edlene Maria Nardes, Fânia Cristina dos Santos

**Affiliations:** 1 Escola Paulista de Medicina, Universidade Federal de São Paulo, São Paulo, SP, Brazil.; 2 Universidade Católica de Pelotas, Pelotas, RS, Brazil.; 3 Centro Universitário Nove de Julho, São Paulo, SP, Brazil.; 4 Hospital Israelita Albert Einstein, São Paulo, SP, Brazil.

**Keywords:** Pain, Dementia, Aged, 80 and over, Pain measurement, Validation studies as topic

## Abstract

**Objective:**

To validate the Pain Assessment Checklist for Seniors with Limited Ability to Communicate – Portuguese in demented elderly and to analyze its measurement properties.

**Methods:**

We evaluated 50 elderly with dementia, residing in a nursing home and with limited communication ability, when exposed to potentially painful situations. The tool was applied at two different moments. First, two interviewers applied it simultaneously, and the intensity of pain was asked based on the caregiver’s opinion. After 14 days, with no analgesic intervention, one of the interviewers applied it again.

**Results:**

The sample comprised more females, aged over 80 years, with dementia due to Alzheimer, presenting musculoskeletal pain of moderate to severe intensity. The psychometric properties of the tool demonstrated appropriate internal consistency (Cronbach’s alpha coefficient of 0.827). The scale had excellent reproducibility, according to the intraclass correlation coefficient, and the tool has been duly validated.

**Conclusion:**

The Pain Assessment Checklist for Seniors with Limited Ability to Communicate – Portuguese had adequate measuring properties for use with elderly presenting limited communication.

## RESUMO

**Objetivo:**

Validar o *Pain Assessment Checklist for Seniors with Limited Ability to Communicate – Portuguese* em idosos dementados e analisar as propriedades de suas medições.

**Métodos:**

Foram avaliados 50 idosos dementados, residentes de uma casa de repouso e com capacidade de comunicação verbal limitada, quando expostos a situações potencialmente dolorosas. A ferramenta foi utilizada em dois momentos diferentes. Primeiro, foi aplicada por dois entrevistadores simultaneamente, e a intensidade da dor foi questionada com base na opinião do cuidador. Depois de 14 dias, sem intervenção de analgésico, a ferramenta foi aplicada novamente por um dos entrevistadores.

**Resultados:**

A amostra teve predominância de mulheres, acima de 80 anos de idade, com demência de Alzheimer, apresentando dores musculoesqueléticas com intensidade moderada a grave. As propriedades psicométricas do instrumento demonstraram consistência interna adequada (coeficiente alfa de Cronbach de 0,827). A escala teve excelente reprodutibilidade, de acordo com o coeficiente de correlação intraclasse, e o instrumento demonstrou validade adequada.

**Conclusão:**

O *Pain Assessment Checklist for Seniors with Limited Ability to Communicate – Portuguese* teve propriedades de medição adequadas para idosos com limitações de comunicação.

## INTRODUCTION

The growth of the elderly population is a universal phenomenon. The projection for 2030 is that individuals aged over 65 years will account for over 20% of population in the United States.^(1)^ Brazil has the fastest aging process in the world, and is estimated to rank sixth in number of elderly citizens in 2025.^(2)^ This epidemiological scenario shows an increase in number of chronic health problems, cancer and disability, and many cases are related to pain.^(3)^

According to epidemiological knowledge, the prevalence of pain increases with age. Its evaluation and management must be prompt and effective. The inadequate control of pain in elderly individuals results in decreased mobility and Activities of Daily Living (ADL), sleep disorders, depression and cognitive impairment. These conditions can be associated with other morbidities, such as deep vein thrombosis, pulmonary embolism, falls, fractures and worsening in quality of life.^(1,4)^ In addition, uncontrolled pain in these individuals could substantially increase healthcare cost.^(5)^

Pain is no longer seen as a simple sensation, and has recently been considered a complex sensory experience, modifiable by one’s memory, expectations and emotions. It presents discriminative, sensory, cognitive, affective and emotional components, considering a subjective experience. It is shaped by the individual context and perception of meaning. It is not easy to address chronic pain in elderly patients, and it is even more difficult in demented patients.

Dementia is one of the main causes of disability and loss of quality of life in elders.^(6)^ The incidence of dementia is expected to rise from 25 million, in 2000, to 114 million, in 2025.^(7)^ The literature shows the prevalence of dementia is 5 to 10% at 64 years, rising to 15 to 20% at 75 years and 40% at 90 to 95 years of age.^(8)^

Potentially painful situations are very common to institutionalized demented elders. The prevalence of pain in these individuals varies from 49 to 83%,^(9,10)^ and is often underdiagnosed and undertreated, especially in those with advanced dementia, who find it difficult to express their pain, making their evaluation more troublesome.^(11-13)^ Studies revealed that 25% of elderly suffering pain did not receive any analgesics, and those aged over 85 years and presenting cognitive impairment received even less treatment.^(14)^

The sensorial perception of pain is usually preserved in elderly. The ability to express pain, nevertheless, could be impaired by cognitive decline or delirium.^(15)^ There are few studies showing that the interpretations of painful stimuli might be altered as a consequence of dementia, and affective response to pain might be increased in demented patients. Other studies suggested these patients are not less sensitive to pain, but less capable of perceiving certain sensations, including pain.^(16,17)^ Thus, elderly individuals with advanced dementia and suffering from pain refer it lesser than those with normal cognition, and receive less analgesics even though they might have the same pain diagnosis.^(10)^

Pain assessment is not easy. Pain is a subjective experience, difficult to quantify and qualify due to the physiological and psychological factors involved. It is necessary to measure pain properly to treat it.

The ability to report pain varies according to the cognitive impairment stage. Mild-to-moderate dementia patients are usually able to answer unidimensional questionnaires and offer self-reports of pain, although they find it difficult to inform the specific site, duration, predisposing and alleviating factors.^(18)^ Moderate-to-severe dementia patients have limitations in communicating and their pain reports are usually inadequate.^(19)^ Behavioral disorders, such as apathy, agitation, vocalization, frowning, antalgic posture, inadequate bed attitudes, pupil dilation and sweating, present as parameters of pain.^(12)^ These reports need to be explored during medical interviews with family members or caregivers.

Thus, pain assessment in cognitively impaired elderly with limited verbal communication is a great challenge in clinical practice. It is very important to review pain history and evaluate its relation to behavioral changes, paying close attention to reports from family and caregivers. Prescribing analgesics empirically might be of help in difficult cases.^(20,21)^

Several tools for assessing pain in elderly patients with advanced dementia have been validated or are under validation process worldwide. Nevertheless, there is no gold-standard tool for this population. In 2002, the American Geriatrics Society (AGS) established broad directives to determine the behavioral indicators of pain.^(22)^ More recently, AGS created the Nurses’ Pain Management Task Force, with the objective of evaluating pain in patients who cannot communicate, including those with dementia. It recommends a comprehensive and hierarchical approach integrating self-reports and behavioral changes.^(23)^

A literature review concerning the psychometric analysis of several pain measurement tools for people with limited communication showed that most of them present fragile validity, reliability and practical use. It also suggested that Pain Assessment Checklist for Seniors with Limited Ability to Communicate (PACSLAC) is an instrument fast to apply with promising qualities.^(24)^

The PACSLAC was specially conceived to evaluate pain in elderly patients with limited communication skills. It is composed of 60 observational items, divided into four different sub-scales: facial expressions, body movements, vocalizations and others. It is quickly applied and easily understood by healthcare professionals.^(25,26)^ The checklist was originally validated in English, but it has been already translated into and validated in French and Dutch.^(24,27)^ In the Netherlands, it is the pain measurement tool most often used by nurses, and it is considered the most promising instrument for pain assessment in dementia.^(24)^ Nurses that routinely use PACSLAC consider it is less stressful and causes no burnout, as compared to others who filled in meaningless checklists.^(28)^

The PACSLAC was translated into Brazilian Portuguese and cross-culturally adapted as the Pain Assessment Checklist for Seniors with Limited Ability to Communicate – Portuguese (PACSLAC-P),^(29)^ but it has not been validated or had its psychometric properties studied.

## OBJECTIVE

To validate the Pain Assessment Checklist for Seniors with Limited Ability to Communicate – Portuguese, with demented elderly with limited communication skills in a nursing home facility, and also evaluate its psychometric measures.

## METHODS

This is a methodological, descriptive and analytical study, approved by the Research Ethics Committee of the *Hospital Israelita Albert Einstein* (HIAE), under protocol CAAE: 07004112.0.0000.0071.

The choice of the participants was based on the convenience sampling; a type of non-probability sampling method that relies on data collection from population members who are conveniently available to participate in study. Thus, the sample was composed of all elderly patients diagnosed with dementia of any kind, according to Diagnostic and Statistical Manual (DSM-IV),^(30)^ with limited communication ability and exposure to (recently or not) potentially painful circumstances (contusions, joint dislocation, fractures, infections/inflammation, and surgery), who lived in *Residencial Israelita Albert Einstein* (RIAE) in the city of São Paulo (SP, Brazil). The subjects were aged ≥60 years, both sexes and of any ethnic group.

Patients who had no formal or informal caregiver to take responsibility for their care, or those who required immediate change or introduction of analgesic treatment prescribed by the attending physician were excluded. A signed Informed Consent was obtained from the legal representatives or, in their absence, from the HIAE administration staff.

Data collection included sociodemographic data, such as sex and age, the probable cause of dementia and pain, and medications taken. Pain was measured using the Visual Analogue Scale (VAS)^(31)^ referred by caregivers who were present daily.

The PACSLAC-P was applied by the researchers (E1 and E2), separately, on the same day, and, in a second moment, after an interval of, at most, 14 days, this instrument was reapplied by one of them (now called E3), making sure that no new analgesic intervention was made in the period.

To study the psychometric properties of PACSLAC-P, reliability and validity were assessed, as recommended for instrument measures.^(32)^ Reliability will be verified accordingly to its internal point consistency (correlations between different items on the test), retest-test reproducibility and inter-rater reliability.

Some methods are used in the validation process, such as face validity (which subjectively evaluates if the instrument measures what it is supposed to do, and that was obtained for PACSLAC-P during its cross-cultural adaptation process);^(29)^ content validity (evaluates if the measurement object is representative; it was also obtained during the cross-cultural adaptation process); construct validity (refers to the general case of translating any construct into an operationalization), criterion-related validity (refers to the degree to which the operationalization is similar to some that it theoretically should be similar to), and others.^(33)^ For this study, the criterion-related validity, convergent type, was obtained. The PACSLAC scores were correlated with scores on the VAS referred by caregivers. A construct validity was not obtained since there was no pain measuring instrument available that was considered gold standard for dementia, at that time.

The softwares Statistical Package for Social Science (SPSS), version 17, Minitab 16 and Microsoft Excel 2010 were used for statistical analysis. The equality of two proportions was tested to characterize the distribution and relative frequency of qualitative variables. The Cronbach’s alpha coefficient was used for internal consistency, and the intraclass correlation coefficient (ICC) and the Kappa coefficient were used for reproducibility. The Pearson’s correlation test was also used in the validation. The significance level was established at 5%.

## RESULTS

The sample was composed of 50 individuals with a mean age of 87.8 years, predominantly women (78%), mostly with dementia due to Alzheimer’s disease (59.2%), frequent use of medications, primarily analgesics, and presenting mainly muscle and joint pain (Table 1).

**Table 1:** Characterization of the sample

Characteristics	n (%)	p value
Age (years)		
Mean (SD) 87.8 (6.5)	50 (100)	
Min-Max 75-100		
Sex		
Female	39 (78)	<0.001
Male	11 (22)	
Type of dementia		
Alzheimer	29 (59.2)	Reference
Vascular	2 (4.1)	<0.001
Mixed	18 (36.7)	0.026
Type of medication		
None	6 (4.3)	<0.001
Antidepressant	24 (17.4)	<0.001
Anticonvulsant	30 (21.7)	<0.001
Analgesic	99 (71.7)	Reference
Antipsychotic	54 (39.1)	<0.001
Vitamin D	72 (52.2)	<0.001
Muscle relaxant	3 (2.2)	<0.001
Cause of pain		
Muscle	132 (89.8)	Reference
Joint	126 (85.7)	0.286
Vascular	54 (36.7)	<0.001
Neuropathic	42 (28.6)	<0.001
Neoplastic	6 (4.1)	<0.001
Ostomy	24 (16.3)	<0.001
Skin lesion	6 (4.1)	<0.001
Other	9 (6.1)	<0.001

SD: standard deviation; Min-Max: minimum and maximum.

In relation to pain intensity according to caregivers, a mean of 60.94mm was observed in the VAS. Thus the sample had mostly moderate pain (Table 2).

**Table 2:** Pain intensity, according to caregivers

Pain		n (%)
VAS (mm)		
Mean (SD)	60.94 (2.24)	
Min-Max	30-100	
Mild	(0-30)	7 (10)
Moderate	(31-70)	31 (44)
Severe	(71-100)	32 (46)

VAS: Visual Analogue Scale; SD: standard deviation; Min-Max: minimum and maximum.

A mean PACSLAC-P score of 3.20 (±0.62) was obtained, and the mean application time was 5 to 7 minutes, which was considered a short period, demonstrating the tool was easily applied and understood.

Relating to its psychometric properties, the internal consistency, as per Cronbach’s alpha coefficient, presented values of 0.646 for facial expressions, 0.619 for body activities/movements, 0.618 for social/personality/mood and 0.247 for others subscale. The PACSLAC-P’s total score was 0.827. Thus, adequate values were observed.

According to ICC, the instrument’s reliability through its reproducibility was considered good or excellent, with 85.2% inter-rater correlation (E2 and E3) and 64.3% in the retest-test correlation (E1 and E3) (Table 3). According to Kappa’s coefficient for variability and reproducibility, the reliability was considered significant (Kappa of 0.381). Therefore, there was relevant inter-rater agreement for all questions of the instrument, but the values were interpreted as just considerable (values between 0.21 to 0.40) (Table 3).

**Table 3:** Reproducibility, according to intraclass correlation coefficient and to Kappa’s coefficient

PACSLAC-P (total score)	E1		E2	
	
	ICC (%)	Kappa	ICC (%)	Kappa
E2	70.7	0.322		
E3	64.3	0.215	85.2	0.381

p value <0.001. PACSLAC-P: Pain Assessment Checklist for Seniors with Limited Ability to Communicate – Portuguese; ICC: intraclass correlation coefficient.

For PACSLAC-P criterion-related validity, adequate values were also obtained. There was a positive and significant correlation in Pearson’s correlation test comparing PACSLAC-P total score and the VAS. The higher was the score in PACSLAC-P, the higher the VAS score, and vice versa (Table 4).

**Table 4:** Validity, according to Pearson’s correlation test

PACSLAC-P (subscales and total score)	Pain intensity	

	Correlation (r) (%)	p value
Facial expressions	58.6	<0.001
Activities/body movements	43.8	0.001
Social/personality/mood	38.6	0.003
Others	58.0	<0.001
Total score	64.3	<0.001

PACSLAC-P: Pain Assessment Checklist for Seniors with Limited Ability to Communicate – Portuguese.

## DISCUSSION

This sample comprised very old individuals (mean age of 87.7 years), who represent a population segment that grows fast.^(23)^ It had a predominance of females (77.6%), which is corroborated by the literature that reports a feminization of aging, especially in the very old group.

Among the types of dementia, Alzheimer’s was the most prevalent, in 59.2% of participants; this is consistent with the literature that reports the condition as the most common cause of dementia.^(34)^ The most frequent answer for probable causes of pain was muscle and joint pain (89.8% and 85.7%, respectively). These data are supported by the literature, which describe bone diseases are conditions that are highly associated with pain by demented elderly patients.^(34)^

The PACSLAC-P was considered easily understandable and of quick application, demanding a short period of time to be answered (5 to 7 minutes). Analyzing the psychometric properties and reliability, according to the internal consistency of each item, very satisfactory results were observed for the total score and subscales, except for the other scale, which had a low result (0.247). Thus, it showed a good internal consistency for the PACSLAC (Cronbach’s alpha coefficient >0.6).

Referring to the reproducibility (inter- and intra-rater reliability), the values were not all high, but were considered adequate. Regarding the ICC, the values were considered good or excellent (85.2%; inter-rater agreement); therefore, according to Kappa coefficient, the values were considerable (0.381; considerable is between 0.21 to 0.40). Thus, one level of reliability was observed.

In addition to the original English version, the PACSLAC was translated and validated into other languages, such as Dutch,^(27)^ French,^(28)^ Korean.^(35)^ In all these versions, the correlation and reliability levels were adequate, likewise the validity levels.

The findings of this study did not differ much from the original PACSLAC.^(24)^ Comparatively, the internal consistency for the total scale was higher (0.82 to 0.92) and the Cronbach’s alpha values for the subscales were lower (0.55 to 0.73) in the original PACSLAC. In the newest validation for Korean (PACSLAC-K), high internal consistency (0.90), and high inter- (0.86) and intra- rater reliability (0.93) were observed.^(29)^

The criterion-related validity was moderate using pain intensity scale based on caregivers’ perception of the patient’s pain as the gold standard. Considering the total score of PACSLAC-P, the correlation was better (64.3%) and the values were adequate. A positive correlation of PACSLAC-P total score and pain measurement through the caregivers’ opinion was obtained, stressing validity of the instrument. In the subcales, the values were lower, ranging between 38.6 and 58,6% (Pearson’s correlation: high >0.7; moderate 0.5 to 0.7; and weak 0.3 to 0.5).

If there were a gold standard assessment test, it would certainly help and enhance the present validity process, and this was one of the limitations of this study. Currently, there are few instruments designed to the same PACSLAC-P purposes available in Brazil. However, when this study was planned, it was not possible to apply other tools and make comparisons. These tools comprise the Pain Assessment Tool in Confused Older Adults (PATCOA),^(36)^ Pain Assessment in Advanced Dementia Scale (PAINAD)^(37)^ and Non-Communicative Patient’s Pain Assessment Instrument (NOPPAIN).^(38)^

It is also important to mention that the sample size was small, but it comprised eligible individuals from a long-term nursing care organization, where the study was carried out.

The PACSLAC and the PAINAD have been the most extensively evaluated tools, with the strongest psychometric properties.^(39)^

Regarding the clinical usefulness of PACSLAC-P, it was also considered a good quality systematic review that examined the psychometric properties of various instruments to measure pain. In Brazil, PACSLAC-P was the first translated and culturally adapted instrument to assess pain in elderly with limited communication skills. This checklist has been used for many years and its psychometric properties are now under investigation. However, further research is needed and the cutoffs points still require validation.

## CONCLUSION

The Pain Assessment Checklist for Seniors with Limited Ability to Communicate – Portuguese proved to be reliable and valid, and it is a very helpful tool to assess and manage pain in elderly patients with limited verbal communication skills. Since it is easy and fast to be applied, it can help healthcare professionals deliver good quality care to elderly with dementia, who may complain of pain.
